# Actively Targeted and Redox Responsive Delivery of Anticancer Drug by Chitosan Nanoparticles

**DOI:** 10.3390/pharmaceutics12010026

**Published:** 2019-12-26

**Authors:** Elisabetta Mazzotta, Selene De Benedittis, Antonio Qualtieri, Rita Muzzalupo

**Affiliations:** 1 Department of Pharmacy, Health and Nutritional Sciences, University of Calabria, Via Savinio, Ed. Polifunzionale, 87036 Arcavacata di Rende, Italy; mazzotta-elisabetta@libero.it; 2 Institute for the Research and the Biomedical Innovation (IRIB)-CNR-Mangone (CS), 00185 Rome, Italy; selene.db90@gmail.com (S.D.B.); antonio.qualtieri@cnr.it (A.Q.)

**Keywords:** redox-responsive, folate-targeting, chitosan nanoparticles, methotrexate, intracellular drug release

## Abstract

The clinical efficacy of methotrexate (MTX) is limited by its poor water solubility, its low bioavailability, and the development of resistance in cancer cells. Herein, we developed novel folate redox-responsive chitosan (FTC) nanoparticles for intracellular MTX delivery. l-Cysteine and folic acid molecules were selected to be covalently linked to chitosan in order to confer it redox responsiveness and active targeting of folate receptors (FRs). NPs based on these novel polymers could possess tumor specificity and a controlled drug release due to the overexpression of FRs and high concentration of reductive agents in the microenvironment of cancer cells. Nanoparticles (NPs) were prepared using an ionotropic gelation technique and characterized in terms of size, morphology, and loading capacity. In vitro drug release profiles exhibited a glutathione (GSH) dependence. In the normal physiological environment, NPs maintained good stability, whereas, in a reducing environment similar to tumor cells, the encapsulated MTX was promptly released. The anticancer activity of MTX-loaded FTC-NPs was also studied by incubating HeLa cells with formulations for various time and concentration intervals. A significant reduction in viability was observed in a dose- and time-dependent manner. In particular, FTC-NPs showed a better inhibition effect on HeLa cancer cell proliferation compared to non-target chitosan-based NPs used as control. The selective cellular uptake of FTC-NPs via FRs was evaluated and confirmed by fluorescence microscopy. Overall, the designed NPs provide an attractive strategy and potential platform for efficient intracellular anticancer drug delivery.

## 1. Introduction

The specific, effective, and safe intratumoral delivery of active drugs is one of the key issues in cancer therapy. Polymeric nanoparticles (NPs) are one of the most promising candidates to achieve this purpose and overcome the drawbacks of traditional chemotherapy. NPs, indeed, gained increasing attention for their efficacy in improving pharmacokinetics and biodistribution drug profiles with a higher degree of selectivity and specificity. NPs have several unique physical and biological properties that allow targeting tumors and minimizing side effects on healthy tissues [1]. The excellent features of NPs include prolonged systemic circulation, high preferential accumulation at the tumor sites via an enhanced permeation and retention effect (EPR), and the ability to overcome P-glycoprotein-mediated multidrug resistance of cancer cells [2]. Among the most commonly used NPs, chitosan (CHT)-based NPs play a pivotal role. CHT is a naturally occurring biopolysaccharide that was extensively explored in pharmaceutical and biomedical fields due to its unique biological activities including antioxidant, anti-inflammatory, antifungal, and antimicrobial activity. This polysaccharide provides a profitable tool for the design of innovative delivery systems due to its biocompatibility, biodegradability, non-toxicity, and mucoadhesivity [3]. Additionally, the cationic nature of chitosan makes it a promising material for drug delivery systems. In fact, the electrostatic interactions with the negatively charged cell surface make this material particularly suitable for pharmaceutical applications. Indeed, the common method used for NP preparation is the ionic gelation technique, an easy method based on the electrostatic interaction between the positively charged chitosan chains and anionic crosslinking agents such as glutaraldehyde, tripolyphosphate, and polyaspartic acid sodium salt [4]. Moreover, CHT is considered a natural anticancer polysaccharide. Many in vitro and in vivo studies were published thanks to the application of this polymer for the design of anticancer tools [5,6,7], but their efficacy is restricted due to the low efficiency of specific targeting [8]. However, the presence of reactive functional groups gives huge opportunities for chemical modifications with a wide range of molecules in order to improve its targeting tumor ability. 

NP, in fact, can be designed to present well-defined properties according to the therapeutic purposes and for their versatility to adapt to the pathological environment and release a drug in a controlled way. Notably, the microenvironment within a tumor is quite different from normal healthy cells, such as its acidic and reductive conditions, different expression levels of some enzymes and receptors, etc. These distinctive features critically affect the success of NPs and, if opportunely considered in the design of nanocarriers, could provide advantages for target cancer treatment. For instance, one important tumor hallmark is the overexpression of specific receptors compared to normal cells [9]. In order to improve nanocarrier selectivity, several approaches were advanced, mostly including the functionalization with ligands such as aptamers, antibodies, proteins, peptides, nucleic acids, or small molecules that specifically bind these receptors and promote cellular uptake via receptor-mediated endocytosis [10,11]. In particular, folic acid (FA) is one of the most employed ligands for the active targeting approach due to its countless advantages such as low cost, high stability, and broad library of conjugation reactions available [12]. In fact, FA can specifically bind to folate receptors (FRs), overexpressed by many kinds of cancer cells; thus, it can be used as a drug-targeting ligand for cancer therapy [13,14]. Various type of folate-based targeting drug delivery systems such as liposomes, nanoparticles, solid lipid nanoparticles, and micelles were designed, and they exerted greater anticancer activity than non-targeted systems [15,16,17,18]. 

Once at the desired site of action, an ideal nanodevice for cancer therapy must still rapidly release the payload to effectively kill tumor cells. To enhance the intracellular drug release, growing interest currently focuses on the design of functional polymers with reactivity to the tumor microenvironment [19].

The incorporation of stimuli-responsive linkages in the polymeric network allows a triggered and localized drug release by responding to endogenous stimuli typical of tumor tissues. Various stimuli-responsive nano-systems such as enzyme-responsive, pH-responsive, temperature-responsive, and redox-responsive systems were developed for controlled delivery applications [20,21,22,23]. Among these, redox potential is of particular importance owing the fact that the concentration of intracellular glutathione (GSH 2–10 mM), a tripeptide responsible for the reduction of disulfide linkages, is approximately 2–3 orders higher than that of the extracellular GSH (2–20 µM) [24]. Furthermore, cancer cells exhibit four-fold higher GSH levels as compared to normal healthy tissues [25] due to the rapid proliferation and the GSH-mediated phase II detoxification mechanism involved in the drug resistance of cancer cells [26]. Accordingly, nano-systems can be tailor-made to be redox-responsive via the incorporation of disulfide linkages thanks to their stability in the physiological environment and their sensitivity in reductive conditions. The reducing environment of tumors plays, thus, the role of an internal signal that allows the destabilization of redox-responsive nanocarriers via the cleavage of the disulfide bond into free thiols. Therefore, the use of redox-responsive NPs is an advantageous method to target the release of a drug inside cells and to consequently improve its efficacy. 

Methotrexate (2,4-diamino-*N*-10-methyl propylglutamic acid, MTX) is a folic acid antagonist widely used in the treatment of autoimmune diseases and different cancers, including acute lymphoblastic leukemia, head and neck cancer, lung cancer, and breast cancer [27,28]. MTX acts by inhibiting dihydrofolate reductase (DHFR), an enzyme involved in DNA and RNA synthesis. However, its clinical efficacy is often compromised by some limitations. Firstly, many undesirable side effects are typically related to MTX. Moreover, its poor solubility in physiological environment and a multidrug resistance mechanism limit MTX efficiency. In order to overcome these limitations, novel MTX delivery systems such as nanoparticles [29], microspheres [30], and liposomes [31] were already developed. The drug delivery in the colloidal systems allows modifying the drugs pharmacokinetics and pharmacodynamics, improving its therapeutic efficacy [32].

The objective of this research was, thus, the design of a multifunctional CHT derivative for targeted delivery of MTX to human cervix adenocarcinoma cells (HeLa). We decided to investigate the antitumor activity of a smart hybrid material simultaneously possessing folate active targeting properties and GSH responsiveness. Firstly, novel folic-thiolated chitosan (FTC) derivatives were synthetized through the conjugation of l-cysteine (l-Cys) and FA to CHT. The resulting polymers were employed for the design of NPs as redox-responsive platforms for the intracellular delivery of MTX. Via folate receptor-mediated endocytosis, the NPs can selectively enter tumor cells and, subsequently, the MTX can be rapidly released in response to the highly reductive environment of the cytoplasm. Therefore, after NP preparation via the ionic gelation technique and physicochemical characterization, release experiments were performed in reductive media mimicking GSH concentrations in extra- and intracellular space. By comparing them with the redox-insensitive CHT-NPs, the in vitro antiproliferative activity of FTC-NPs was studied on HeLa cells using the 3-(4,5-dimethylthiazol-2-yl)-2,5-diphenyltetrazolium bromide (MTT) assay. Furthermore, the cellular uptake efficiency was evaluated by fluorescence microscopy. All studies were carried out to evaluate the potential of the nano-systems proposed as devices for efficient intracellular MTX delivery.

## 2. Materials and Method

### 2.1. Chemicals

In this study, we used the following chemicals: 5,5-dithiobis(2-nitrobenzoicacid) (DTNB, Ellman’s reagent), MTX, methotrexate; CHT, chitosan at low molecular weight; TPP, sodium triphosphate; PBS, phosphate buffer solution; HCl, hydrochloric acid; NaBH_4_, sodium borohydride; FA, folic acid; FRs, folate receptors; EDC, 1-ethyl-3-(3-dimethyllaminopropyl) carbodiimide hydrochloride; MTT, 3-(4,5-dimethylthiazol-2-yl)-2,5-diphenyltetrazolium bromide. 

### 2.2. Preparation of Folic-Thiolated Chitosan

**Figure 1:**
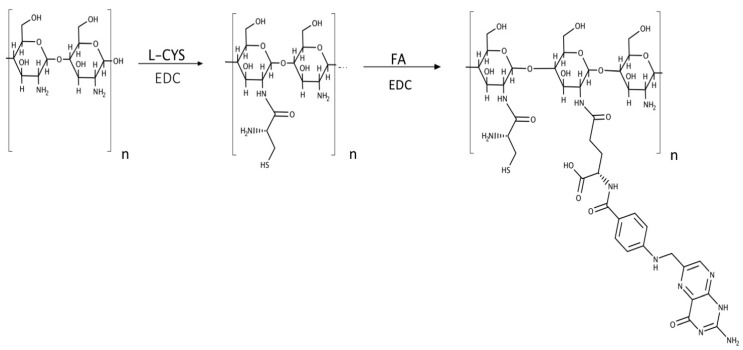
Synthesis route of folic-thiolated chitosan. Abbreviations: l-CYS, l-cysteine; EDC, 1-ethyl-3-(3-dimethyllaminopropyl) carbodiimide hydrochloride; FA, folic acid.

The synthesis of folic-thiolated chitosan (FTC) was carried out as shown in Figure 1 via the conjugation of l-Cys and FA to the CHT backbone in a two step-process. Specifically, derivatives with three different weight ratios of CHT and l-Cys (1:1, 1:2, 1:4) were synthesized, and the quantities used are reported in Table 1.

Briefly, CHT was dissolved in acetic acid 1% (*v*/*v*) to obtain a 1% (*w*/*v*) polymer solution. Separately, the carboxylic group on l-Cys was activated by EDC in demineralized water for 1 h and then added dropwise to the above solution under magnetic stirring. The pH of the reaction mixture was carefully adjusted to 5 with NaOH 0.5 M. This mixture was stirred for 4 h in the dark at room temperature. After that, the reaction mixture was extensively dialyzed (molecular weight (MW) cutoff 12 kDa) for three days, firstly against 5 mM HCl, then twice against 5 mM HCl containing 1% NaCl, and finally against 1 mM HCl. At the end, the polymers were lyophilized and stored at 4 °C until further use.

The FA conjugation was performed using the following method: briefly, EDC (0.04 mmol) was added to 2 mg∙mL^−1^ folic acid (0.02 mmol) in dimethyl sulfoxide and stirred at room temperature for 1 h. Separately, 100 mg of thiolated-chitosan derivative was dissolved in 10 mL of acetic acid 1% (*v*/*v*), added to the above solution and stirred in the dark at room temperature for 16 h. At the end of the reaction, the mixture was precipitated, changing the pH to 9. The precipitate was collected and then dialyzed (molecular weight cutoff, 12 kDa) against an excess amount of 0.1 M sodium phosphate buffer (pH 7.4) for three days and then water for another three days. The resulting products were freeze-dried and subjected to ^1^H nuclear magnetic resonance (NMR) characterization to demonstrate the successful conjugation of l-Cys and FA to the CHT. ^1^H-NMR (300 MHz, D_2_O) δ (ppm): 1.75 (s, –NHCOCH_3_), 2.55 (s, –CH_2_SH), 2.87 (s, proton of glucosamine unit of CHT), 3.30–3.76 (m, proton of glucosamine unit of CHT), 7,17 (d, aromatic proton of FA), 7.56 (d, aromatic proton of FA), 8.51 (s, aromatic proton of FA). 

### 2.3. FTC Characterization

#### 2.3.1. Ellman Test

The number of free thiol groups on FTC conjugates was determined with Ellman’s reagent according to a previously reported method [33]. Briefly, 0.5 mg of each conjugate was hydrated in 500 µL of 0.5 M phosphate buffer pH 8.0. Then, 500 µL of 0.03% (*w*/*v*) DTNB dissolved in the same buffer was added to the sample, and the resulting solution was incubated, protected from the light, for 2 h at 37 °C. Finally, the absorbance of the supernatant was measured at 405 nm, and the number of thiol groups was calculated on the basis of a standard curve obtained using l-Cys. The content of disulfide bonds was measured after reduction with NaBH_4_ and determined with Ellman’s reagent as described above.

#### 2.3.2. Quantification of FA

The amount of FA on novel designed polymers was evaluated using ultraviolet–visible light (UV–Vis) JASCO V-530 spectrometer (Jasco Europe SRL, Como, Italy), considering its absorption at 285 nm. The FTC conjugates were dissolved in 1% acetic acid at the concentration of 2 × 10^−2^ mg/mL, and absorbance measurements of these solutions were performed. The FA concentration was calculated on the basis of a standard curve previously plotted from a stock solution of FA dissolved in a 0.1 M NaOH solution, from which subsequent dilutions in 1% acetic acid were made in the range 0.2–2 × 10^−2^ mg/mL. 

### 2.4. Nanoparticle Preparation

FTC nanoparticles were prepared according to the ionic gelation technique which is based on the complexation of an anion crosslinking agent, sodium triphosphate (TPP), with a cationic polymer. Briefly, FTC polymers were dissolved in acetic acid 1% (*v*/*v*) achieving a concentration of 3 mg/mL. Then, 0.5 mL of MTX alkaline solution (2.02 × 10^−2^ M) was added, and the pH of the resulting solution was adjusted to 5.5 with NaOH 1 M. After that, NPs were immediately and spontaneously formed by adding dropwise 1 mL of aqueous solution of TPP (2 mg/mL) under constant stirring at room temperature. The purification of the resulting NPs was carried out by ultracentrifugation at 15,000 rpm for 90 min.

### 2.5. Nanoparticle Characterization

The size and distribution of the NPs were determined, at 25.0 ± 0.1 °C, by dynamic light scattering (DLS) analysis using a 90 Plus Particle Size Analyzer (Brookhaven Instruments Corporation, New York, USA). The samples were analyzed 24 h after preparation with a dilution ratio of 1/100 in distilled water, and the data were elaborated with the Contin method [34]. Each sample was measured six times, and the results were expressed as means ± standard deviation (SD). The zeta potential values of NPs were determined using the Zeta-sizer ZS (Malvern Instruments Ltd., Malvern, U.K.), at 25.0 ± 0.1 °C. The measurements were obtained after a dilution of 1/50 in distilled water, and the data were reported as the means of three independent experiments performed in triplicate. The morphology and size of the NPs were analyzed using transmission electron microscopy (TEM) (Jeol 1400 Plus electron microscope, JEOL ltd., Milano, Italy) after treatment with a 2% phosphotungstic acid solution.

### 2.6. Drug Entrapment Efficiency

MTX encapsulation efficiency was determined via an indirect method measuring the amount of untrapped drug. Briefly, NPs were centrifuged at 15,000 rpm for 90 min in order to separate the non-encapsulated drug from NPs, and then the amount of free MTX in supernatant was analyzed using UV spectrophotometry at 306 nm. The entrapment efficiency was calculated according to the following equation:

(1) $$EE\;\%=\frac{MTX\;tot-MTX\;free}{MTX\;tot}\;\times \;100.$$

Additionally, drug loading (DL) was calculated by the following equation:

(2) $$DL\;\%=\;\frac{Entrapped\;MTX\;\left(wt\right)}{Inital\;polysaccharide\;\left(wt\right)}\;\times \;100.$$

### 2.7. In Vitro Redox-Responsive Drug Release

MTX release of NPs was carried out in phosphate buffer pH 7.4 in the absence and presence of 10 mM GSH to simulate the physiological conditions and reductive cytoplasm of cancer cells, respectively. 

Briefly, an NP aliquot was dialyzed at 37 °C and, at set time intervals, 2 mL of the medium was taken and analyzed using UV–visible light spectrophotometry at 306 nm. All experimental procedures were repeated three times, and the results were in agreement within ±4% standard error.

### 2.8. Cell Culture

HeLa cells from the American Type Culture Collection (ATCC, Manassas, VA, USA) were grown in Dulbecco’s modified Eagle’s medium (DMEM) (Thermo-Fischer Scientific, Waltham, MA, USA) supplemented with 10% (*v*/*v*) fetal bovine serum (FBS), 2 mM l-glutamine, 100 UI/mL penicillin, and 100 μg/mL streptomycin at 37 °C in a humidified incubator with 5% CO_2_. The cells in 25-cm^2^ flasks were passaged into 96-multiwell plates, using trypsin ethylenediaminetetraacetic acid (EDTA), when they reached approximately 80% confluence.

### 2.9. In Vitro Cytotoxicity Assay

The in vitro cytotoxicity of the blank and MTX-loaded NPs was evaluated in HeLa cells using the MTT assay. Briefly, the cells (8 × 10^4^/mL) were allowed to settle by incubating the plates for 24 h at 37 °C and 5% CO_2_. After the addition of 0.1, 1, and 10 µg/mL free MTX, MTX-loaded FTC-NPs, and MTX-loaded CHT-NPs, the cells were incubated for 4 h and, subsequently, washed and further cultured with fresh medium up to a total of 24, 48, and 72 h. Untreated HeLa cells were used as a normal control. Moreover, the cytotoxicity of empty NPs toward the cells was evaluated under identical conditions. At the end of the incubation time, 100 μL of MTT (5 mg/mL) dissolved in Dulbecco’s phosphate-buffered saline, pH 7.4, was added to each well and, after an additional 4 h of incubation at 37 °C and 5% CO_2_, the medium was removed and a solubilization solution (100 μL, 16% SDS in 40% dimethylformamide (DMF), pH 4.7, prepared at 37 °C) was added to dissolve the formazan crystal, followed by incubating the plate for another 30 min at 37 °C. An ELX800 microplate reader (Bio-Tek Instruments, Inc., Winooski, VT, USA) was used to measure, in triplicate, at 570 nm the amount of formazan product. The percentage of viable cells was calculated using the following equation:

(3) $$cell\;viability=\frac{AT}{AU}\;\times \;100,$$

where AT is the absorbance of the treated cells and AU is the absorbance of the untreated cells. Cell viability values were expressed as the means of at least three different experiments ± SD.

### 2.10. Cell Uptake Studies 

Sodium fluorescein (NaFl)-loaded FTC-NPs were prepared using the same methods described above (for MTX) and then purified by ultracentrifugation at 15,000 rpm for 90 min. NaFl was loaded to provide a final concentration of 0.03 mg/mL. Hela cells were seeded onto six-well plates on a coverslip in DMEM (medium) and incubated overnight before the addition of NaFl-loaded CHT-NPs (100 μL/mL). Free NaFl added to DMEM (0.003 mg/mL) was used as a control. Upon incubation at 37 °C for 4 h, the media were removed, and then the cells were washed with PBS three times and fixed with 4% paraformaldehyde in PBS for 10 min at room temperature. All coverslips were mounted on clean glass slides with UltraCruz, a mounting medium containing 4′,6-diamidino-2-phenylindole (DAPI0 (Santa Cruz biotechnology, TX, USA), and examined on a conventional fluorescent microscope, Nikon Microphot EPI-FL, (Nikon, JP), equipped with a mercury lamp.

### 2.11. Statistical Analysis

Student’s *t*-test was used for statistical analysis and *p*-values ≤ 0.05 were considered statistically significant.

## 3. Results

### 3.1. FTC Characterization

New multi-functional CHT-based NPs possessing redox responsiveness and folate-targeted properties were developed and investigated for potential application in tumor target therapy. Nanocarriers responsive to the reductive conditions are particularly appealing for intracellular drug delivery applications in cancer therapy. Notably, chitosan, one of the biodegradable polymers most used for tumor drug delivery, was selected as a starting material due to the presence of reactive amino groups that make it an attractive biomaterial apt to several modifications in order to improve tumor targeting ability. 

In this study, l-Cys and FA were functionalized and conjugated to the CHT backbone through amide bonds in a two-step reaction. EDC was used as a coupling agent, and three different derivatives with various CHT/l-Cys *w*/*w* ratios of 1:1, 1:2, and 1:4 were synthetized. 

These modifications had many advantages. The first was that l-Cys residues can be easily oxidized by air to give inter- and intramolecular disulfide bonds that are cleavable in a reductive environment. Consequently, the incorporation of disulfide linkages into the NP structure made them redox-responsive, leading to a prompt and selective drug release only in response to reductive stimuli in a specially controlled manner. On the other hand, the FA was employed to functionalize NPs for active tumor targeting and to allow a preferential accumulation on tumor tissue via FR-mediated endocytosis. The combined activity of FR targeting and redox responsiveness is proposed in order to achieve an improved MTX delivery in cancer cells and, thus, high therapeutic efficacy. 

The synthetic pathway of the FTC polymer is summarized in Figure 1, and the chemical structure was characterized by ^1^H-NMR analysis. The NMR spectra of synthesized FTC indicated a new peak at δ 2.75 ppm corresponding to methylene protons of l-Cys, confirming the conjugation reaction and the successful formation of a thiol-functionalized polymer. Moreover, the coupling of FA residues with CHT was confirmed by the appearance of the peculiar signals at 7.17, 7.56, and 8.51 ppm, which are characteristic peaks attributed to the aromatic protons of the folate ring. The successful conjugation of FA was also confirmed by UV–visible spectroscopy analysis and the amounts of FA conjugated are given in Table 1.

The numbers of free thiol groups and disulfide bonds immobilized on FTC were determined using the Ellman test, and the results are shown in Table 1. FTC2 polymer exhibited the highest disulfide content (92.29%) among all polymers designed, followed by FTC3 (70.71%) and FTC1 (65.87%).

### 3.2. Nanoparticle Characterization

**Figure 2:**
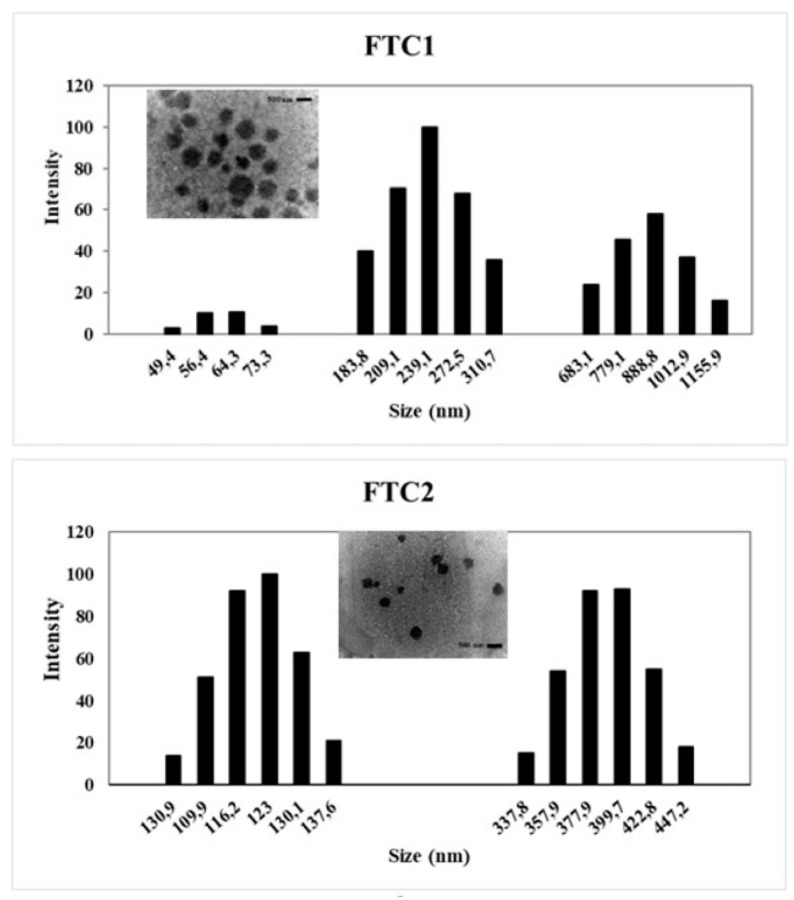
Size distribution and TEM micrographs (bar 500 nm) of nanoparticle (NP) folic-thiolated chitosan samples FTC1 and FTC2.

Glutathione-responsive NPs were obtained via the ionic gelation technique based on the complexation of positively charged FTC polymers with a polyanion crosslinking agent [35]. All the formulations developed were homogeneous and really stable over long periods (more than four weeks, at room temperature and in the dark). The morphological features of NPs were evaluated by TEM. TEM pictures (Figure 2) revealed the presence of well-defined spherical NPs with a smooth surface.

Physicochemical characteristics are important parameters from a pharmaceutical viewpoint, and their evaluation plays a crucial role in predicting the NP in vivo stability. Consequently, NPs developed in this work were characterized in terms of particle size, polydispersity index (PI), shape, and zeta (Z)-potential, and the results are shown in Table 2.

NPs had a narrow size distribution as highlighted by PI values lower than 0.3, as reported in Table 2. The mean hydrodynamic diameters ranged between 202 nm and 378 nm, in good agreement with those evaluated by TEM analysis.

These small sizes (<400 nm) make them suitable for use as drug delivery carriers. The particle size, in fact, affects the drug release characteristics and the uptake in tumor tissues. In the literature, it is widely reported, indeed, that solid tumors show a hypervascular permeability and impaired lymphatic drainage; thanks to this, NPs can significantly accumulate in tumors via the EPR effect that typically operates in the range of 100–400 nm [36].

Commonly, the presence of disulfide intermolecular linkages in the polymer structure is shown to affect the NP size [37]. The increase in disulfide content allows the formation of smaller devices. This trend conforms to the present experiments; in fact, smaller NPs were obtained with FTC2 polymers owing to their higher disulfide content compared to other polymers and plain CHT. DLS measurements of loaded samples demonstrated that, comparing to empty samples, no significant variation in particle size occurred after drug loading. 

Formulations investigated in this research showed positive zeta-potential values ranging between +24.9 ± 1.4 and +35.9 ± 0.36 mV, suggesting a high colloidal stability. Moreover, their cationic nature makes them effective devices for anticancer drug delivery to solid tumors. The positively charged nanoparticles can promote high cellular uptake efficacy owing to the electrostatic interactions with cellular anionic components [38]. 

The MTX entrapment efficiency values ranged from 18.28% to 55.92%. The three different derivatives showed different drug incorporation capacity. The reasons for that behavior are still obscure and could probably be ascribed to different interactions between the drug and polymeric matrix.

### 3.3. In Vitro Release Studies

A good stability in blood circulation and the ability to rapidly and thoroughly release the drug into the intracellular environment of cancer cells are important aims to be achieved for the design of ideal drug delivery systems for tumor therapy. 

**Figure 3:**
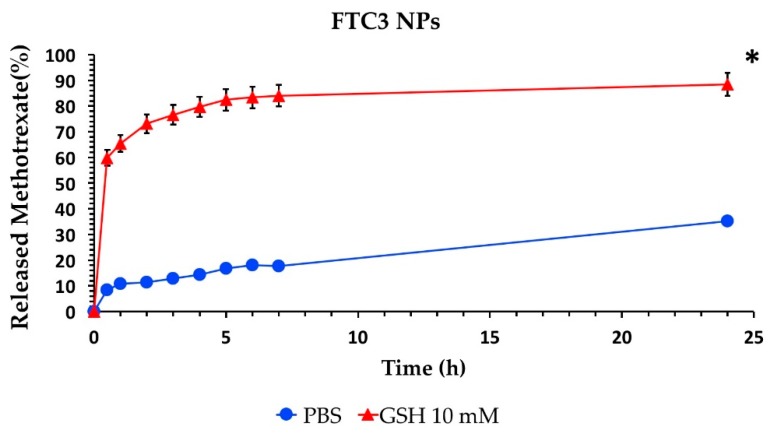
In vitro drug release profiles from methotrexate (MTX)-FTC3 NPs in phosphate buffer in the presence (▲) and absence (●) of glutathione (GSH) 10 mM at 25 °C (mean ± standard deviation, *n* = 3). The amount of MTX release in phosphate buffer in the presence of GSH 10 mM at every time point was statistically different (* *p* < 0.05) from that recorded in the absence of GSH.

To verify the redox-responsive properties of the prepared NPs, the release studies were carried out at pH 7.4 PBS in the presence and absence of 10 mM GSH to mimic the tumor tissue and physiologic environment, respectively. As is known, GSH concentration in the intracellular environment of tumor cells is about four-fold higher than in normal healthy cells. Hence, GSH was chosen as the reducing agent to evaluate the redox-responsive nature of NPs. Drug release profiles for the FTC3-NP sample are given in Figure 3. A slow release of below 35% of MTX within 24 h was observed in PBS at pH 7.4, indicating a good stability in the extracellular medium of healthy tissues and only a little drug leakage during blood circulation. Oppositely, in the presence of 10 mM of GSH, a faster drug release took place owing to the breakage of the crosslinking points in the NP structure, exhibiting a release equal to 59.85% in the first 30 min and up to 88.4% in 24 h. These results demonstrated that the cleavage of disulfide bridges led to a rapid and complete drug release. Redox-responsive NPs have, indeed, a high stability in bloodstream. On the other hand, FTC nanoparticles can be rapidly disassembled by GSH in the highly reductive cytoplasm of the tumor, which results in a quick MTX release and an effective inhibition of tumor growth. 

**Figure 4:**
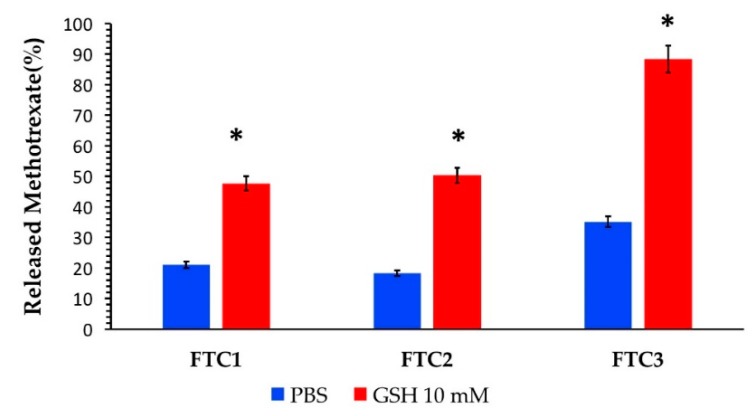
Percentage of MTX released from NPs based on FTC polymers with different amounts of disulfide content (FTC1 65.87%; FTC2 92.29%; FTC3 70.71%) in phosphate buffer in the presence and absence of GSH 10 mM at 25 °C (mean ± standard deviation, *n* = 3). The amount of MTX release in phosphate buffer in the presence of GSH 10 mM was statically different (* *p* < 0.05) from that recorded in the absence of GSH.

The influence of disulfide content on release was also investigated, and the results are summarized in Figure 4. Previously, Hu et al. [39] reported that the redox reaction is a rate-dependent phenomenon, and the reduction-responsive ability could be modulated by varying the amount of disulfide content depending on the application desired. In our studies, we also found that the drug release profile was closely dependent on the number of disulfide bonds present in the polymer structure, confirming the earlier studies. A higher degree of disulfide content in NPs is related to more high responsiveness. In fact, FTC2 proved to be the polymer with the highest number of disulfide bonds, which led to a 2.8-fold higher drug release in the presence of a reducing agent compared to that in physiological conditions. The redox sensitivity decreased for FTC3 and FTC1 with 2.5- and 2.2-fold increases in drug release, respectively, related to the lower crosslinking point in the NP structure. 

Overall, these results confirm our expectations, since the nanocarriers were considerably stable before reaching the tumor sites but achieved complete and accelerated release inside tumor cells. In fact, the in vitro drug release experiments confirmed the GSH responsivity of the FTC nanocarrier and support their great potential for intracellular tumor drug delivery.

### 3.4. In Vitro Cytotoxicity

**Figure 5:**
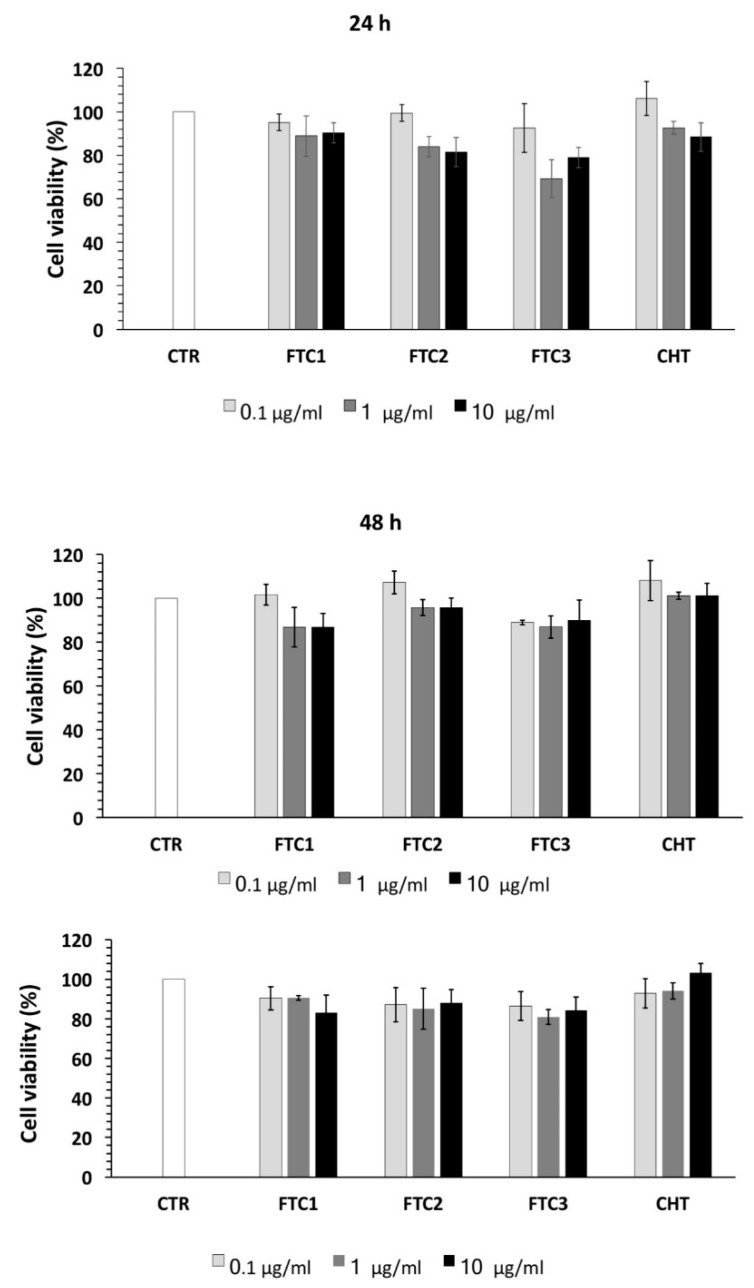
HeLa cell viability tested by 3-(4,5-dimethylthiazol-2-yl)-2,5-diphenyltetrazolium bromide (MTT) assay in triplicate. Cells were incubated with blank (control, CTR), and FTC- and CHT-based NPs at different concentrations for 24, 48, and 72 h. The results are expressed as the percentage of the control assumed as 100%. Each value represents the mean ± SD of three independent experiments.

In order to study the anticancer activity of designed formulations, cell viability was evaluated using the MTT assay in HeLa cells after being exposed to free MTX, MTX-FTC NPs, and MTX-CHT NPs at different concentrations for 24, 48, and 72 h, as showed in Figure 5. The results showed that cell viabilities remained higher than 80% after 72 h of incubation with empty systems in the range of concentrations investigated, indicating the biocompatible and non-toxic nature of FTC polymers. 

**Figure 6:**
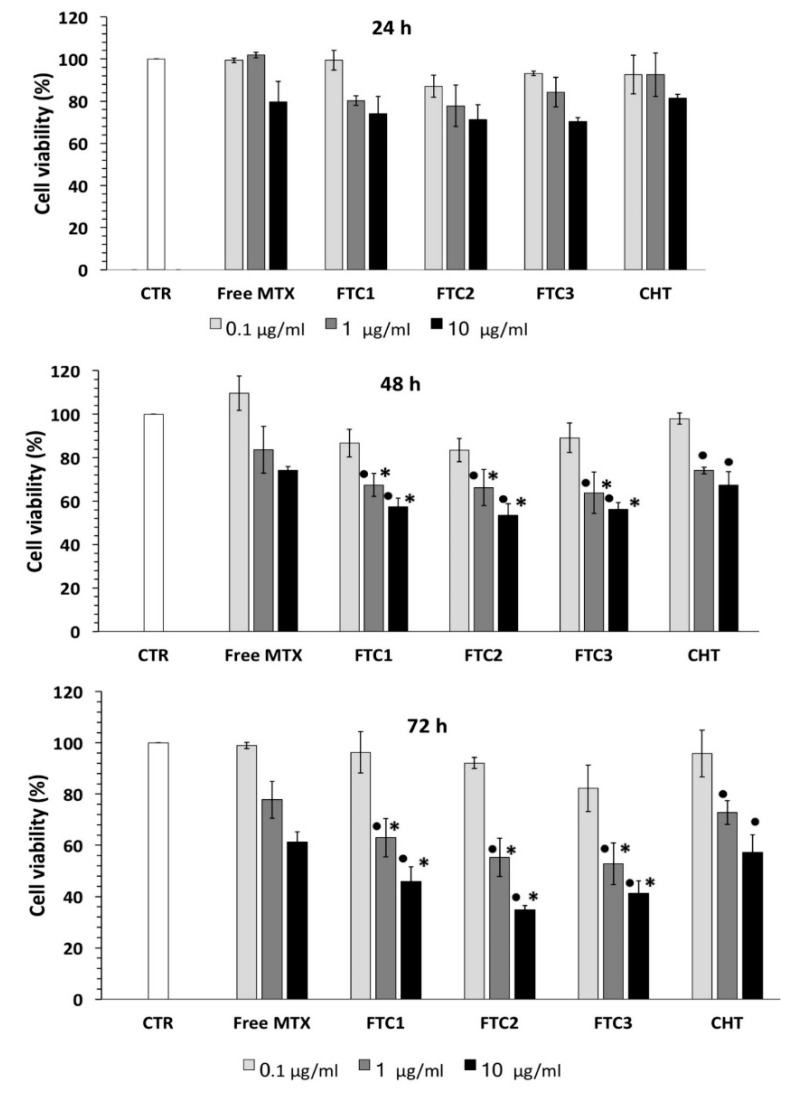
HeLa cell viability after incubation with free MTX, MTX-loaded FTC-NPs, and MTX-loaded CHT-NPs at 0.1, 1, and 10 μM for 24, 48, and 72 h evaluated using the MTT assay. The results are expressed as the percentage of the control assumed as 100%. Each value represents the mean ± SD of three independent experiments. • *p* < 0.05 vs. control; * *p* < 0.05 vs. CHT-coated liposome-treated cells.

On the contrary, a good cell growth inhibition effect was observed for drug-loaded systems. As shown in Figure 6, the encapsulation of MTX in NPs greatly improved its anticancer efficacy and affected the cell viability in a time- and dose-dependent manner. 

Both MTX-FTC NPs and MTX-CHT NPs, in fact, showed higher cytotoxicity toward HeLa cells than free MTX, maybe because the drug-loaded NPs could enter the cell more selectively compared to free MTX. Typically, MTX clinical efficacy is limited by the appearance of resistance in cancer cells [40]. The enhanced cytotoxicity of MTX nano-sized carriers could be due to the improved drug cell internalization via endocytosis and lower drug efflux from cells [41]. Furthermore, our studies indicated that the cytotoxicity of MTX-FTC NPs was higher than that of the non-target MTX-CHT NPs used as a control. Specifically, the cell survival rate was reduced to 57.28% after treatment for 72 h with MTX-loaded CHT NPs, while the values were 45.92%, 34.81%, and 41.23% with the use of FTC1, FTC2, and FTC3 NPs, respectively. The increased cytotoxicity of MTX-FTC NPs highlighted that the redox-responsive and folate-targeting NPs had better potential for MTX intracellular delivery in contrast with the non-target NPs.

This may be attributed to the high affinity of folate-modified NPs to tumor cells and a quick intracellular release of MTX under stimulation of GSH in the cytoplasm of cancer cells. Thus, FTC-NPs may be a promising solution for delivering chemotherapeutic drugs to tumors, but their potential needs to be confirmed through in vivo tests.

### 3.5. Evaluation of FTC-NP Uptake Using Fluorescence Microscopy Analysis

**Figure 7:**
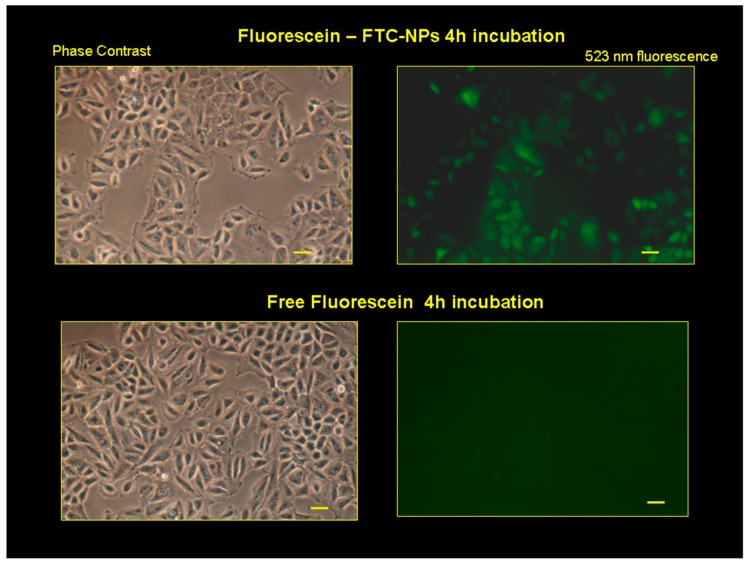
Fluorescence microscopic pictures of HeLa cells after incubation over 4 h with culture medium containing NaFl-loaded FTC-NPs and free NaFl. The fluorescence excitation was at 475 nm, and detection was at 523 nm. The respective phase contrast images are also shown. Scale bar = 50 μm.

To evaluate the cellular uptake ability of the designed devices in this work, green fluorescent NaFl was loaded into the FTC-NPs. The evaluation of the internalization ability of nanocarriers is essential for predicting the in vivo performance. Consequently, evidence of cellular uptake was obtained by fluorescence microscopy analysis performed in HeLa cells after treatment with free NaFl and NaFl-loaded FTC-NPs for 4 h. As seen in Figure 7, nearly no green fluorescence was observed in HeLa cells incubated with free NaFl, which was due to the low permeability of fluorescein in the cell membrane [42]. Conversely, the fluorescence of HeLa cells was greatly enhanced after treatment with FTC-NPs. This result showed that FTC-NPs were effective in the enhancement of NaFl uptake. This behavior can be attributed to FR’s mediated endocytosis of NPs. Our results are, thus, in agreement with previous cytotoxic studies, according to which FTC-NPs were more effective than free drug in the inhibition of cancer cell growth. From these results, FA-decorated redox-responsive NPs are able to enhance the intracellular release and to target drug selectivity in tumor cells.

## 4. Conclusions 

In this study, multifunctional CHT nanoparticles with folate-targeted and redox-responsive properties were successfully developed as intracellular MTX delivery systems for improved antitumor activity. To achieve this goal, disulfide bonds were introduced into the CHT backbone, endowing a desirable redox sensitivity to this system for triggering a selective and localized intracellular drug release in the tumor microenvironment; the inclusion of folate ligands, instead, could enhance the selectivity to tumor cells. The FTC-NPs exhibited an excellent physiological stability and a significantly faster drug release in the reductive environment. the in vitro antitumor activity demonstrated that multifunctional NPs possess a better anticancer activity in contrast to the non-targeted NPs. Consequently, the designed NPs hold great promise as nanocarriers for targeted cancer therapy, and further in vivo studies must be conducted in this field to prove their efficacy.

## Figures and Tables

**Table 1:** Amounts of reagents used (±10%) for the synthesis of folic-thiolated chitosan (FTC) conjugates with different weight ratios of l-cysteine (l-Cys) and results of the number of thiol groups and folic acid (FA) content obtained. EDC—1-ethyl-3-(3-dimethyllaminopropyl) carbodiimide hydrochloride; CHT—chitosan; Fol—folate.

Conjugate	Chitosan (mg)	l-Cys (mg)	EDC (mM)	CHT:Cys:Fol Ratio (*w*:*w*:*w*)	–SH Total μmol/g Polymer	% Disulfide Bond	Folic Acid μmol/g Polymer
**FTC1**	150	150	50	1:1:0.1	114.0 ± 5.3	65.87	5.60 ± 0.28
**FTC2**	150	300	150	1:2:0.1	407.5 ± 9.5	92.29	5.05 ± 0.41
**FTC3**	150	600	150	1:4:0.1	141.9 ± 7.0	70.71	5.11 ± 0.18

**Table 2:** Average size, polydispersity index, zeta (Z)-potential, entrapment efficiency, and drug loading of nanoparticles (NPs) at 25 °C. Data are mean values ± SD (*n* = 3). PI—polydispersity index; MTX—methotrexate; EE—encapsulation efficiency; DL—drug loading.

Formulation	Size (nm)	PI	Z-Potential (mV)	EE% MTX	DL %
FTC1	364.2 ± 3.1	0.167	+28.3 ± 1.04	-	
FTC2	202.4 ± 5.8	0.254	+35.9 ± 0.36	-	
FTC3	234.7 ± 7.9	0.234	+24.9 ± 1.4	-	
CHT	378.4 ± 7.4	0.318	+30.7 ± 3.35	-	
FTC1-MTX	363.9 ± 3.3	0.154	+26.3 ± 1.18	55.92 ± 2.88	18.64 ± 0.28
FTC2-MTX	258.3 ± 4.9	0.153	+28.9 ± 0.47	37.44 ± 5.22	12.48 ± 0.52
FTC3-MTX	302.0 ± 9.3	0.250	+26.8 ± 1.89	18.28 ± 9.87	6.09 ± 0.99
CHT-MTX	364.1 ± 2.5	0.273	+26.7 ± 0.99	55.15 ± 4.52	18.38 ± 0.45
